# Digital model for monitoring national programs: the Kazakhstan experience

**DOI:** 10.3389/frai.2025.1656329

**Published:** 2025-11-19

**Authors:** Mafura Uandykova, Tolkyn Mirkassimova, Gulnar Mukhamejanova, Assel Yeleukulova, Akhmed Baikhojayev, Gulnar Astaubayeva

**Affiliations:** 1 School of Digital Technologies, Narxoz University, Almaty, Kazakhstan; 2 Kazmetengineering LLP, Almaty, Kazakhstan

**Keywords:** digital government, national program monitoring, e-governance, predictive analytics, public administration, performance evaluation

## Abstract

This paper presents a conceptual digital model for monitoring national programs designed to enhance their effectiveness, transparency, and performance in the context of digital transformation in public administration. The research identifies limitations of traditional monitoring approaches characterized by data fragmentation, lack of dynamic tracking, and insufficient focus on socio-economic outcomes. In response to these challenges, we propose an original Digital Model for National Program Monitoring (DMNPM) that integrates various data sources from Kazakhstan’s digital ecosystem (egov.kz, Smart Bridge, Open Data). The key scientific contribution of the model is its comprehensive approach, which includes predictive analytics capabilities based on machine learning for risk forecasting and causal relationship assessment, as well as built-in two-way feedback mechanisms. To demonstrate the practical applicability and potential of DMNPM, we present case studies of monitoring key strategic programs in Kazakhstan – “Digital Kazakhstan” and “Nurly Zhol,” as well as pilot national projects “Zhaily Mektep” and “Auyldyq Densaulyq Saqtau.” A quasi-experimental pilot across two national programs demonstrates measurable improvements in monitoring effectiveness and reporting efficiency compared to traditional manual processes. The research contributes to digital governance theory and monitoring methodology by offering a practical solution adapted for countries with actively developing digital infrastructure.

## 1 Introduction

In the context of accelerated digitalization of public administration, the issue of effectiveness and transparency in the implementation of national programs becomes critically important. Despite significant investments in e-government development and the implementation of platform solutions (such as egov.kz, Smart Bridge, Open Data), traditional monitoring approaches in Kazakhstan, as in many other countries, face fundamental problems. These problems include data fragmentation, lack of real-time performance tracking mechanisms, focus on formal reporting rather than actual socio-economic results, and low adaptability to rapidly changing conditions. As a result, this impedes timely, evidence-based management decisions and reduces the overall effectiveness of government initiatives.

The digital transformation of government has progressed from early “e-Government” initiatives focused on online service delivery to more integrated “Digital Government” approaches that leverage data analytics, machine learning, and platform ecosystems to transform internal processes and create more responsive, citizen-centered services (Janowski, 2015; Mergel et al., 2019). Recent studies demonstrate that the human-machine fit and regulatory oversight significantly impact trust in these digital systems (Li et al., 2025), while the implementation of generative AI introduces both efficiency gains and epistemological risks (Cugurullo and Xu, 2024). However, the monitoring of national programs has not evolved at the same pace, with many countries still relying on retrospective, paper-based systems that fail to leverage the potential of digital technologies.

The use of artificial intelligence in government faces the complex dynamics of policies that counteract algorithmic formalization and inherent limitations in determining relationships with (Fischer-Abaigar et al., 2024). This requires hybrid monitoring systems that combine machine learning capabilities with collective intelligence technologies with expert participation to comprehensively evaluate the program. This study addresses the following research question: how can a comprehensive digital model improve the efficiency, transparency and effectiveness of national program monitoring in the context of digital government transformation?

The aim of this research is to develop and substantiate a conceptual digital model for monitoring national programs to improve their effectiveness, transparency, and performance in the context of digital transformation of public administration in the Republic of Kazakhstan. To achieve this aim, the following objectives are addressed: analyzing current limitations of traditional monitoring approaches; studying international best practices; developing a conceptual architecture for a Digital Model for National Program Monitoring; substantiating the integration of existing government information systems; demonstrating the model’s potential on specific national programs; and formulating implementation recommendations.

This research fills an existing gap in academic literature and practice by proposing an original conceptual DMNPM, developed considering the specifics and accumulated experience of digitalization of public administration in the Republic of Kazakhstan. The novelty of the model lies in its comprehensive approach, which for the first time in the context of Kazakhstan’s practice integrates disparate data sources with advanced analytical tools (see Supplementary Table S1)such as predictive analytics based on machine learning. While the current pilot implementation utilizes foundational statistical methods (see technical documentation link after Supplementary Table S1), the model’s architecture is designed to accommodate advanced machine learning capabilities as historical data accumulates—a critical consideration given insufficient historical data for robust ML model training in government systems across developing economies (United Nations, 2024).

Analysis of key theoretical and empirical research in public administration, digitalization, and government program monitoring reveals current trends, existing gaps, and positions this research accordingly. Discussions about improving the efficiency and transparency of public administration are central to modern science. The transition from “traditional bureaucracy” (Weber, 1978) to “New Public Management” (Hood, 1991; Osborne and Gaebler, 1992) emphasized the need to implement market principles and result-orientation. However, as information technologies developed, the focus shifted toward “E-Government” (Dawes, 2008; Fountain, 2001) and, later, “Digital Government” (Criado and Ruvalcaba-Gomez, 2022).

Digital government, unlike simply transferring services online, implies a comprehensive transformation of internal processes, the use of big data, artificial intelligence, and platform solutions to create seamless, user-oriented services and proactive governance (Janowski, 2015; Gil-Garcia et al., 2018). Recent studies show that digitalization can significantly improve public sector efficiency (World Bank, 2021), enhance interaction between citizens and government (United Nations, 2024), and contribute to achieving Sustainable Development Goals (UNDP, 2022).

However, success of digital transformation depends not only on technology implementation, but also on institutional reforms, organizational culture changes, and development of appropriate legislation (Al-Busaidy and Weerakkody, 2011; Grindle, 2007).

Monitoring and evaluation (M&E) are fundamental components of effective public governance, providing feedback for decision-making and accountability (Bovens et al., 2014; Aristigueta et al., 2014). Traditional M&E models typically based on periodic reports, selective checks, and retrospective analysis are criticized for: information delays (data collected and analyzed with delays, hindering timely program adjustments); data fragmentation (information often stored in disparate departmental systems); process rather than outcome orientation (many M&E systems focus on measuring inputs and activities rather than actual socio-economic results); and low transparency (limited access to data and reports). In response to these challenges, the academic community and international organizations (OECD, 2021; World Bank, 2021; UNDP, 2022) are actively exploring the potential of digital technologies to transform M&E. Concepts of “Digital M&E” and “Big Data M&E” propose using big data capabilities, analytics, and automation to obtain more accurate, timely, and comprehensive information (UNDP, 2022; World Bank, 2021).

Research shows that open data platforms can significantly increase transparency and civic participation in monitoring (Bertot et al., 2010; Janssen et al., 2012), while data integration through interagency platforms (as in Estonia’s X-Road; Kitsing, 2011) is a key success factor. However, recent analysis reveals that user satisfaction and perceived value are the actual drivers of continued OGD usage, rather than mere availability (Liang et al., 2025). In recent years, there has been an increase in research on applying digital tools in government program monitoring, including the use of: integrated information systems for consolidating data from various sources (Kim and Kim, 2020); dashboards and data visualization for providing intuitive and operational reports (Matheus et al., 2020); geographic information systems for spatial analysis of project implementation (Clarke et al., 2017); and predictive analytics for forecasting future trends and identifying risks (Kankanhalli et al., 2019; Sæbø et al., 2011).

However, most existing research either focuses on individual aspects of digital M&E or describes fragmentary cases. Insufficient attention is paid to developing comprehensive, integrated digital models that consider the specifics of national digital ecosystems and include the full cycle from data collection to proactive decision-making and feedback.

Analysis of the literature shows that, despite growing interest in digital M&E, there is a clear research gap regarding the development and testing of holistic digital models for monitoring national programs that: provide deep integration of disparate government information systems; include advanced analytical capabilities; offer a comprehensive feedback mechanism; and specifically consider the experience of countries with actively developing digital infrastructure.

Our study aims to fill this gap by proposing an original conceptual Digital Model for National Program Monitoring that integrates unique aspects of Kazakhstan’s digital ecosystem with advanced analytical methods, contributing significantly to both academic theory and public administration practice.

## 2 Research methodology

This research is based on methods aimed at developing and conceptually substantiating a digital model for monitoring national programs. The research had a descriptive-analytical character with elements of design science research focused on creating an artifact – the proposed digital model. The research was conducted within a qualitative research approach using the Republic of Kazakhstan as a case study. The choice of Kazakhstan as a case is due to the active development of digital infrastructure for public administration, the presence of key platforms such as egov.kz, Smart Bridge, and Open Data, which creates a unique environment for analysis and development of an applicable digital model.

For forming the conceptual basis of the model and its substantiation, several methods of information collection and analysis were used: *documentary analysis* (studying official strategic documents of Kazakhstan concerning digitalization, e-government systems, and monitoring); *review and synthesis of academic literature* (in-depth analysis of scientific articles on e-government, monitoring and evaluation of government programs, and application of big data in the public sector); and *systematic analysis of existing information systems* (studying architecture and functional capabilities of key government platforms in Kazakhstan). For empirical validation, we also analyzed media reports, survey data, and expert assessments, applying appropriate validation and bias correction procedures for each source type.

The development of the DMNPM was carried out iteratively, based on the synthesis of the obtained data, using conceptual modeling to develop the general architecture, comparative analysis to compare the proposed model with existing global approaches, and scenario analysis to demonstrate the model’s applicability on examples of national programs.

For evaluating the effectiveness of national programs and the added value of the digital monitoring model, a three-level evaluation framework was developed based on systematic comparison of established strategic planning methodologies. Analysis of strategic planning methods reveals distinct approaches: SWOT Analysis focuses on strengths/weaknesses and opportunities/threats but lacks quantitative measurement capabilities for ongoing monitoring; PEST Analysis examines Political, Economic, Social, and Technological factors but emphasizes environmental scanning rather than performance measurement; Balanced Scorecard (BSC) offers Financial, Customer, Internal Process, and Learning & Growth perspectives, with its learning dimension informing our K3 component; Cognitive models provide sophisticated cause-effect mapping but require extensive expert input unsuitable for routine monitoring.

Our three-level framework combines quantitative rigor, outcome orientation, and broader impact perspective:

1. *K1 - Budget efficiency*: Measures the efficiency of budget utilization, including the percentage of allocated funds spent according to plan, the cost–benefit ratio, and the timeliness of financial transactions.
2. *K2 - Goal achievement*: Evaluates the degree to which the stated objectives of the program are met, including quantitative targets and qualitative indicators of success.
3. *K3 - Socio-economic effect*: Assesses the broader impact of the program on society and the economy, including indirect benefits, long-term sustainability, and contribution to strategic national goals.

Each component was evaluated on a standardized scale (0–100%), allowing for comparative analysis across different programs and time periods. The analysis focused on demonstrating DMNPM’s data integration capabilities and identifying systematic limitations in traditional monitoring approaches.

### 2.1 Limitations

This study has several important limitations. We note that this is a concept-led study with a limited pilot; nationwide implementation and a full multi-site evaluation remain future work. The emphasis on Kazakhstan’s experience limits direct generalization of conclusions without additional adaptation to other national contexts. The study does not consider detailed technical specifications, cybersecurity systems, or the complexity of system integration that may arise during a specific implementation. In addition, the study highlights public and academic perspectives, primarily with the limited participation of citizens and civil society organizations suffering from implementation.

## 3 Results

**Figure 1:**
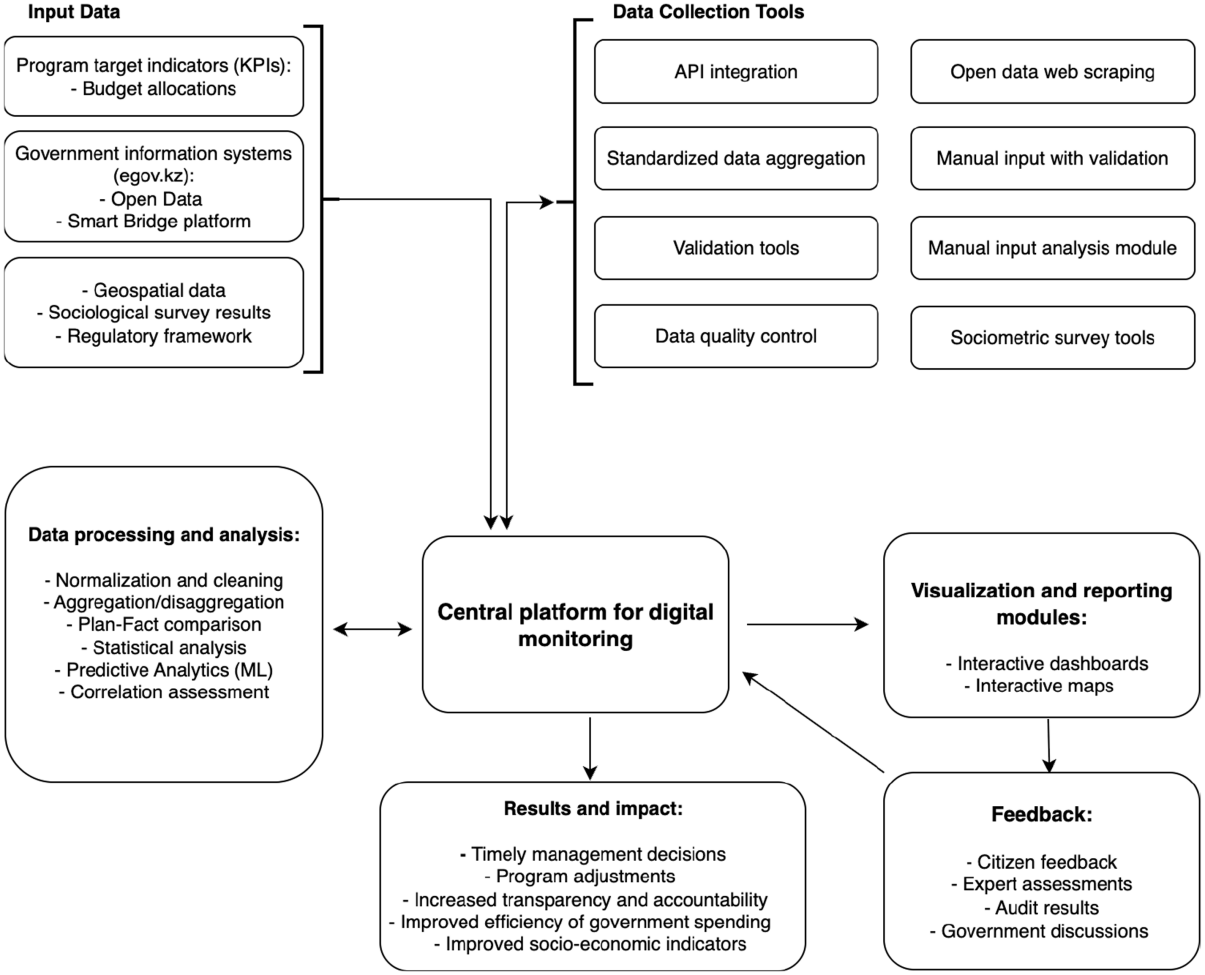
Architecture of the digital model for monitoring national programs.

To ensure transparency, efficiency, and effectiveness in the implementation of national programs in the context of digital transformation, we propose a conceptual DMNPM (see Figure 1). This model aims to overcome the limitations of traditional approaches by integrating modern digital technologies, analytical tools, and feedback mechanisms to provide dynamic, data-driven control and decision-making. The proposed DMNPM is based on key principles including result orientation (focus on measuring actual socio-economic results); real-time monitoring (using digital platforms for dynamic data collection and analysis); data integration (combining disparate data sources); transparency and accountability (ensuring information accessibility to stakeholders); predictive analytics (using analytical tools to forecast potential deviations); and iterativeness and adaptability (flexible adjustment of the model).

As shown in Figure 1, the DMNPM architecture includes the following key components:

- *Input data:* The foundation of the model, including program target indicators (KPIs), budget allocations, data from various government information systems (including egov.kz), open data, Smart Bridge platform data, geospatial data, sociological survey results, and regulatory framework.
- *Data collection modules*: Responsible for automated and standardized aggregation of information. Include API integration with government information systems, automatic collection from Open Data, web scraping (if necessary), manual input module with validation, and tools for sociometric surveys.
- *Central platform for digital monitoring*: The core of the system, providing centralized storage, processing, and management of all collected data. Acts as an integration hub between all components.
- *Data processing and analysis modules*: Transform raw data into meaningful information. Include data normalization and cleaning, aggregation/disaggregation, “Plan-Fact” comparison, statistical analysis, predictive analytics (ML), and correlation assessment to identify trends and anomalies.
- *Visualization and reporting modules*: Provide visual and accessible presentation of monitoring results. Offer interactive dashboards (for different stakeholders), interactive maps, report generator, notification and alert system, and open access to aggregated data.
- *Results and impact*: The ultimate goals and benefits of implementing the model, including timely management decisions, program adjustments, increased transparency and accountability, improved efficiency of government spending, and improved socio-economic indicators.
- *Feedback*: A continuous process of receiving information from citizens, experts, audits, and from discussions in government bodies. This feedback is used for continuous improvement of both the model itself and the national programs being implemented, closing the management cycle.

**Figure 2:**
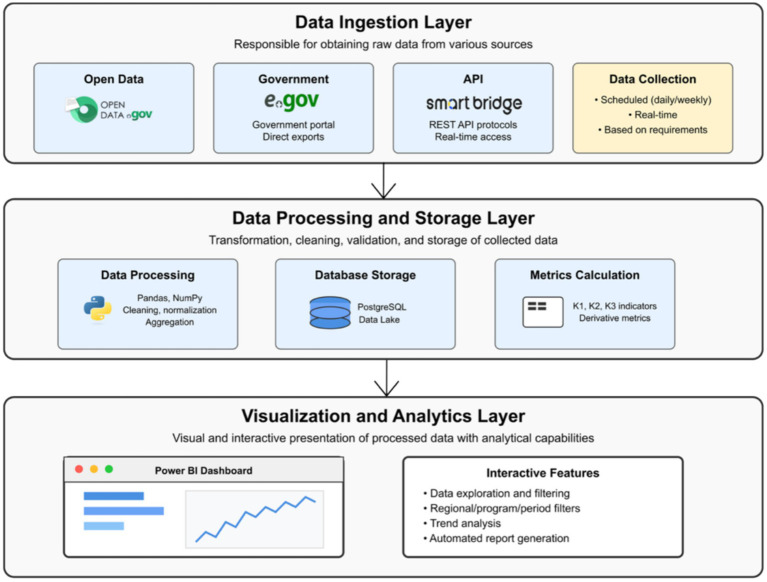
Levels of the digital monitoring model architecture.

The architecture of the digital monitoring model is built on three main levels that ensure a complete cycle of data work:

1. Data ingestion layer: This level is responsible for obtaining raw data from various sources. As shown in Figure 2, the main sources are Open Data (data.egov.kz), egov.kz, and, if access is available, Smart Bridge API. For publicly available data, Python parsers or direct exports are used. For Smart Bridge API, standard interaction protocols (e.g., REST API) are assumed for programmatic data extraction. Data collection can be scheduled (e.g., daily, weekly) or in real-time, depending on source availability and information relevance requirements.
2. Data processing and storage layer: At this level, transformation, cleaning, validation, and storage of collected data take place. “Raw” data often contain gaps, duplicates, or incorrect formats. Using Python (Pandas, NumPy), operations of cleaning, normalization, aggregation, and transformation of data into a structure suitable for analysis are performed. Processed data can be stored in relational databases (e.g., PostgreSQL) for structured data or in non-relational databases/file storages (e.g., Data Lake) for semi-structured and unstructured data. This level also calculates complex indicators such as K1, K2, K3, and any derivative metrics needed for monitoring.
3. Visualization and analytics layer: This level is responsible for presenting processed data in a visual and interactive format, as well as for providing analytical capabilities. Data quality is enforced via an automated pipeline (completeness, consistency, timeliness checks), with flagged records routed to manual review. Microsoft Power BI is used as the main platform for visualization. Interactive dashboards are created, allowing users to explore data, filter it by various parameters (region, program, period), and view trends. Reports can be configured for regular generation and distribution to interested parties.

**Figure 3:**
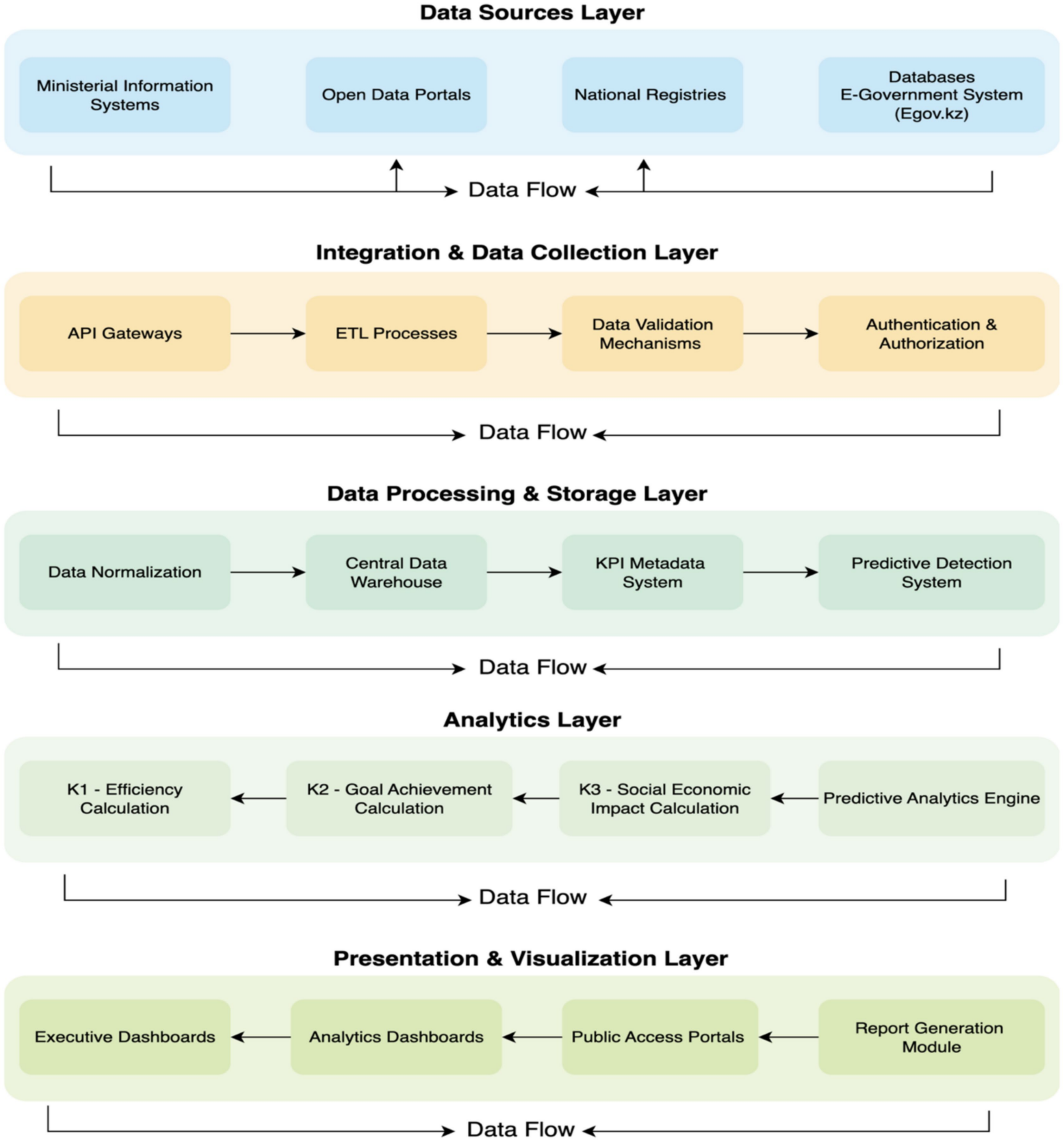
KPI integration with government information systems.

One of the key aspects of the model is the deep integration of key performance indicators (KPIs) of national programs with external government platforms (see Figure 3):

As shown in Figure 3, if access is provided, integration through Smart Bridge API will allow direct receipt of up-to-date data on KPI implementation from departmental information systems. This ensures maximum efficiency and minimizes manual input, reducing the likelihood of errors. KPIs published on the open data portal will be regularly parsed and integrated into the model. This ensures transparency and allows the use of publicly available official data for validation and supplementation of information obtained from other sources. Mechanisms for matching and unifying KPIs from various sources are being developed to ensure a unified representation and avoid duplication or contradictions.

The model implements an automated feedback system that includes deviation triggers (setting threshold values for K1–K3), generation of notifications in case of deviations exceeding thresholds (email, messengers, built-in panels), and forecasting indicators based on linear regression and time series (statsmodels, scikit-learn). This multi-layered approach has proven successful in various contexts, including China’s digital government projects across 31 provinces (Gong and Yang, 2025).

**Figure 4:**
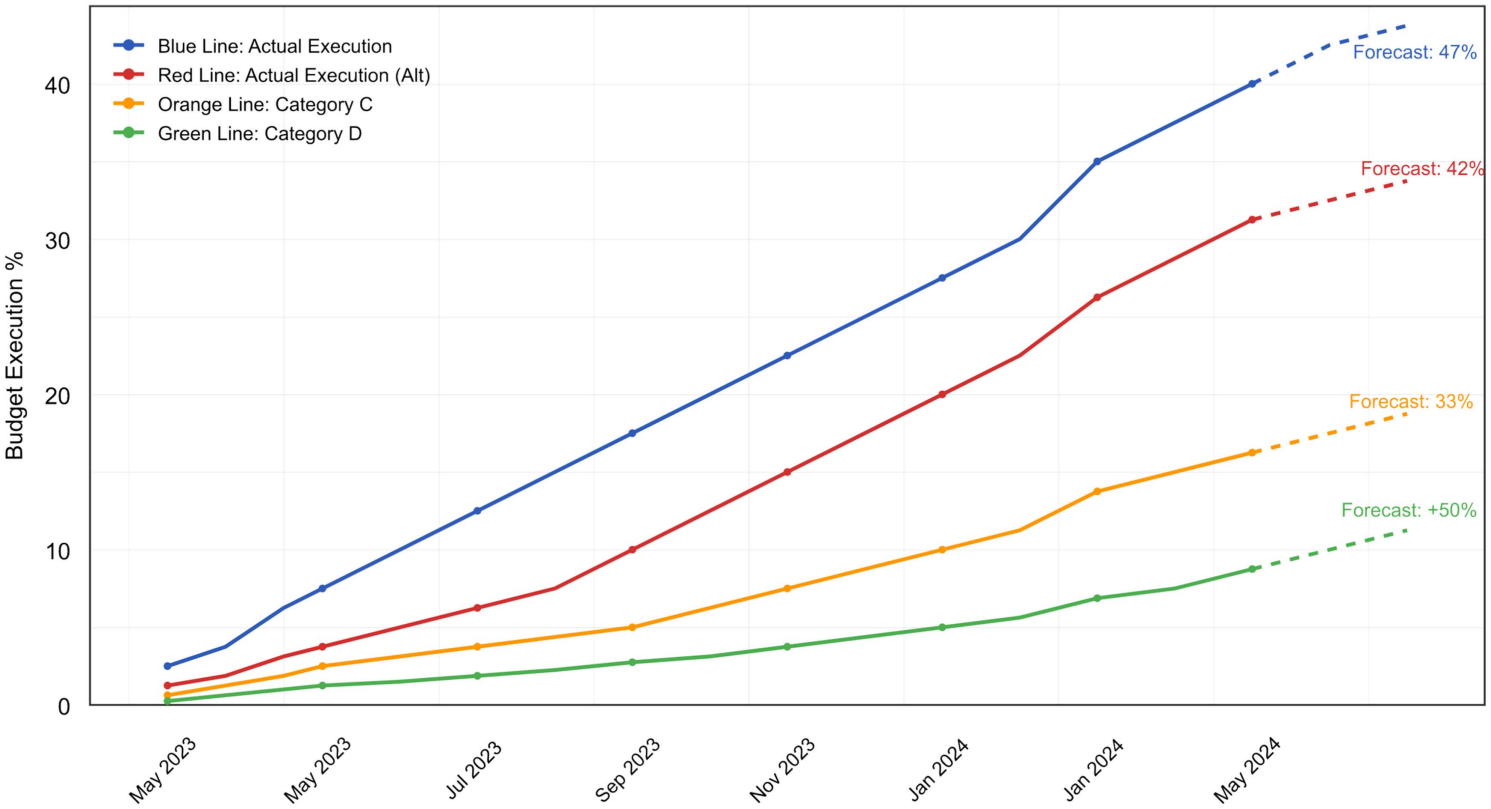
Example of automated forecasting using historical data.

Figure 4 shows an example of automated forecasting of budget execution based on historical data. Different colored lines represent various budget components or categories, allowing comparison of their dynamics and projected changes. This system allows for prompt response to problems before they reach a critical phase and informs responsible executors in real-time. The violation accounting system maintains a log of events and builds a chronology of departmental responses, which is critically important for transparency.

The presented Digital Model for National Program Monitoring offers a comprehensive approach to assessing the progress and effectiveness of government initiatives. Its advantages become particularly evident when analyzing the implementation of strategic programs such as “Digital Kazakhstan” and “Nurly Zhol,” as well as pilot projects “Zhaily Mektep” and “Auyldyq Densaulyq Saqtau,” where traditional monitoring methods often face problems of data fragmentation and delays.

For validation of the developed digital monitoring model in conditions close to real ones, an empirical study was conducted based on two priority national projects of the Republic of Kazakhstan: “Zhaily Mektep” (Comfortable School) and “Auyldyq Densaulyq Saqtau” (Rural Healthcare). These projects were chosen due to their strategic importance, implementation scale, social orientation, and potential availability of data on key indicators.

Zhaily Mektep aims to solve the problem of three-shift education, emergency schools, and shortage of student places by building new comfortable schools throughout the country. The project is characterized by significant budget infusions, complex construction logistics, procurement, and the need for coordination between central and local executive bodies. Monitoring in this case required tracking construction timelines, budget utilization, commissioning of facilities, as well as the impact on the accessibility and quality of education.

Auyldyq Densaulyq Saqtau aims to improve the accessibility and quality of medical care for rural residents, including the construction and modernization of medical facilities, equipping them with equipment, as well as training and attracting medical personnel to villages. The project involves monitoring the network of medical institutions, staffing, service accessibility, and, ultimately, the impact on rural population health indicators.

For each of these projects, a detailed analysis of data collected using the methodology described in Section 3 was conducted, including information from Open Data, egov.kz, and other available sources (see Table 1).

**Table 1:** National project funding volumes (2021–2025).

National project	Funding amount (billion tenge)	Share in total funding (%)
Agro-industrial complex development	1,010.2	11.4
Quality and accessible healthcare	783.3	8.8
National spiritual revival	21.8	0.2
Improving education quality (“Educated Nation”)	185.6	2.1
Technological breakthrough	415.8	4.7
Entrepreneurship development	1,460.0	16.5
Quality infrastructure (“Powerful Regions”)	2,248.6	25.4
Sustainable economic growth, employment	3,022.4	34.1
Green Kazakhstan	157.6	1.8
TOTAL	8,862.9	100.0

Source: Data of the Republic of Kazakhstan (Bureau, 2025).

As seen from Table 1, national projects receive significant funding, with the largest share (34.1%) allocated to the sustainable economic growth and employment project, followed by the quality infrastructure development project (25.4%). Projects related to the social sphere, such as healthcare and education, receive a relatively smaller share of funding, which may require special attention to the efficiency of allocated funds usage.

The application of the developed three-level evaluation model (K1 - Budget Efficiency, K2 - Goal Achievement, K3 - Socio-Economic Effect) allowed for a comparative analysis of the selected cases and identification of strengths and weaknesses of each project, as well as general trends and differences in their implementation in terms of efficiency and impact:

1. *K1 (Budget Efficiency):* The dynamics of budget funds utilization was analyzed. The “Zhaily Mektep” project demonstrated consistently high budget utilization (~90–95% execution across most regions). At the same time, the “Auyldyq Densaulyq Saqtau” project faced greater heterogeneity in execution (from 60 to 100%), especially in remote areas, indicating specific logistical and administrative challenges.
2. *K2 (Goal Achievement):* The assessment was focused on the fulfillment of key indicators. For the “Zhaily Mektep” project, target indicators for school construction were clearly fixed in the form of quantitative metrics (e.g., number of schools commissioned, number of student places created), which significantly facilitated progress monitoring. In the case of the “Auyldyq Densaulyq Saqtau” project, goals were often described more generally, which reduced the accuracy of measuring actual performance, despite obvious progress in improving infrastructure.
3. *K3 (Socio-Economic Effect):* This is the most complex level of analysis. Both projects faced difficulties in collecting direct qualitative data on long-term impact. However, the application of the digital model allowed visualizing and comparing indirect indicators, such as growth in population coverage of services, reduction in complaints about accessibility, as well as acceleration of facility commissioning timeframes, indicating a positive socio-economic effect, although requiring further detailed assessment.

**Figure 5:**
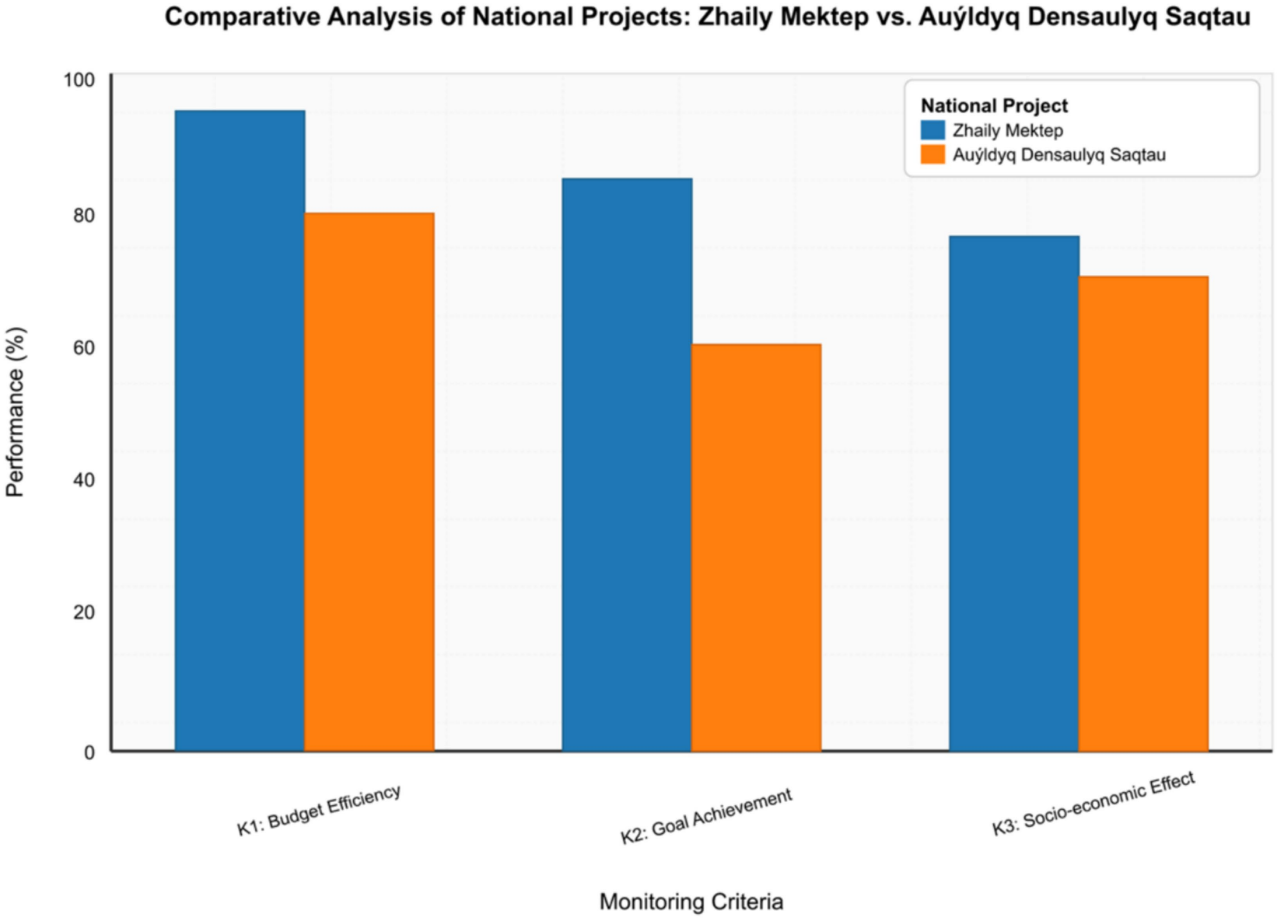
Comparative analysis of national projects by K1-K3 criteria.

As shown in Figure 5, the “Zhaily Mektep” project shows higher results in all three criteria compared to the “Auyldyq Densaulyq Saqtau” project. The largest gap is observed in goal achievement (K2), which may be related to clearer quantification of target indicators in the first project.

Analyzing the data in Table 2, significant differences in budget execution between various national projects can be identified. The highest percentage of budget execution is observed in the projects “Educated Nation” (100%), “Quality and accessible healthcare” (99.8%), and “National spiritual revival” (97.5%), while the least effective in terms of budget execution were the projects “Green Kazakhstan” (35.1%) and “Sustainable economic growth, employment” (36.3%). Such a spread of indicators points to the need for detailed analysis of the reasons for low execution and development of corrective measures.

**Table 2:** Data on national project implementation.

No	National project name	Number of indicators (in document)	Number of activities	Funding (billion tg)	Number not completed	Budget: allocated	Executed	Deviation	Execution (%)
1	Agro-industrial complex development (2021–2025)	22 indicators, 77 activities	21 in document	1,010.2	1 indicator	933.6	-	−76.8	92.4%
2	Quality and accessible healthcare	21 indicators, 73 activities	21 in document	783.3	–	781.3	-	−1.9	99.8%
3	National spiritual revival	20 indicators, 66 activities	20 in document	21.8	1 indicator, 1 activity	21.2	-	−0.5	97.5%
4	Improving education quality (“Educated Nation”)	15 indicators, 26 activities	15 in document	185.6	–	185.6	-	0	100%
5	Technological breakthrough	24 indicators, 120 activities	24 in document	415.8	2 indicators, 1 activity	206.3	-	−209.5	49.6%
6	Entrepreneurship development	30 indicators, 206 activities	29 in document	1,460.0	7 indicators, 18 activities	1,197.9	-	−262.1	82.0%
7	Quality infrastructure (“Powerful Regions”)	31 indicators, 121 activities	30 in document	2,248.6	7 indicators, 7 activities	2,193.1	-	−55.4	97.5%
8	Sustainable economic growth, employment	30 indicators, 115 activities	28 in document	3,022.4	4 activities	1,096.7	-	−1,925.7	36.3%
9	Green Kazakhstan	19 indicators, 51 activities	19 in document	157.6	–	55.3	-	−102.3	35.1%
	TOTAL	206 indicators, 736 activities	190 in document	8,862.9	15 indicators, 44 activities	5,928.6	-	−2,634.3	69.2%

Source: Report on the execution of the republican budget of the government of the Republic of Kazakhstan (2023).

The results of the comparative analysis of empirical cases confirm the viability and functionality of the developed DMNPM in real conditions. The model demonstrated its ability to aggregate heterogeneous data and provide a comprehensive picture of project implementation, identifying both successes and problem areas. The differences in efficiency between the “Zhaily Mektep” and “Auyldyq Densaulyq Saqtau” projects according to the K1-K3 criteria emphasize the importance of: clear quantification of goals (projects with more measurable goals are easier to digitally monitor and evaluate); model flexibility (the ability of DMNPM to adapt to different levels of data detail and various challenges); and significance of indirect indicators (in cases where direct measurements are difficult, the digital model allows using and visualizing indirect indicators for socio-economic effect assessment).

This practical analysis demonstrates how DMNPM enables the transition from formal tracking to deeper, dynamic, and result-oriented monitoring, providing timely information for program adjustment. Comparative analysis also demonstrated that a high level of budget execution does not always correlate with goal achievement or with real social effect, especially without the integration of feedback.

## 4 Discussion

This study presents a conceptual DMNPM designed to enhance the efficiency, transparency, and effectiveness of government initiatives in the context of digital transformation of public administration. This section synthesizes the key findings of the research, analyzing the proposed model in the context of existing academic discussions and international experience, comparing its strengths with limitations, and assessing the potential for scaling.

The DMNPM we have developed marks a significant step in the evolution of government program monitoring methodology, particularly for countries with actively developing digital infrastructure. When compared with the experience of digital governance leaders such as Estonia, South Korea, and Canada, the unique contribution and potential of our model become evident.

While Estonia’s X-Road system (Kitsing, 2011) demonstrates almost perfect seamless interagency integration, Kazakhstan’s infrastructure, including Smart Bridge, faces historical data fragmentation. Our model, considering these realities and offering integration mechanisms on top of existing differences, is a pragmatic and valuable solution for similar conditions in other developing countries, differing from idealized models applicable to already consolidated digital ecosystems.

Furthermore, South Korea actively integrates artificial intelligence and big data for proactive public administration (Kim and Kim, 2020). The inclusion of a predictive analytics module in the model’s architecture based on machine learning in our DMNPM represents a substantial step from reactive to proactive monitoring, allowing not just to record deviations but to predict them, enabling preventive measures (Kankanhalli et al., 2019). This is a critically important distinction from many traditional monitoring systems and positions DMNPM as an advanced solution.

Canadian experience, with its emphasis on “data-driven government” and open data publication, resonates with our approach to using Open Data portals in Kazakhstan. However, Canada demonstrates more advanced mechanisms for engaging citizens in analyzing and using this data to increase accountability (Bertot et al., 2010). Our model, providing open access to aggregated data and interactive dashboards, makes an important step in this direction, but also indicates the need for further development of civic participation culture in Kazakhstan.

Supplementary Table S3 provides a structured comparison of DMNPM with Estonia’s X-Road, South Korea’s Digital Platform Government, and Canada’s GC InfoBase across architecture, technology maturity, AI/analytics capabilities, transparency, and cost efficiency. Key takeaways: DMNPM balances Estonia’s interoperability lessons with Korea’s AI integration, while addressing middle-income country constraints through modular design and pragmatic tool selection.

DMNPM currently implements foundational security mechanisms suitable for government infrastructure: OAuth 2.0 with role-based access control and multi-factor authentication (MFA), TLS 1.3 encryption, database pseudonymization, OWASP API (OWASP Foundation, 2023), and 7-year audit logging with SIEM integration. Operator oversight is mandatory for budget allocations exceeding 10 million tenge. AI is used only for auxiliary functions (semantic search, document processing) without directly influencing resource allocation decisions. When expanding functionality, we plan to follow the OECD AI Principles (OECD, 2024) and the EU AI Act (European Commission, 2024) governance framework for high-risk public sector applications.

While DMNPM includes statistical forecasting with expansion roadmap for advanced analytics, it is important to recognize that AI models work with formal parameters while human decision-making involves cognitive semantics—the conceptual meanings that exist in human consciousness rather than just objective reality (Boldyrev, 2014; Zokirova, 2021). As Zokirova (2021) emphasizes, lexical meaning in cognitive semantics is conceptual, relating to notions in consciousness rather than necessarily to real objects. This requires integration with collective intelligence approaches—the combined capacity of groups to solve shared problems through collaborative technologies (Lowry et al., 2023; Berditchevskaia et al., 2023).

The practical implementation of the model can benefit from integration with existing situational centers established in Kazakhstan since 2011. Janenova (2018) documents that situational centers were created for online video monitoring of all population service centers (ЦОНs), while the Government of Kazakhstan (2020) confirms these centers operate under presidential directives for real-time monitoring. Integration with this existing infrastructure, including the current Unified Situational Center, would accelerate DMNPM implementation through established visualization and coordination mechanisms.

The consideration for DMNPM implementation is compliance with the long-term strategic framework of Kazakhstan, in particular the strategy “Kazakhstan-2050.” The model should include many temporary criteria: short-term indicators that measure the operational effectiveness of the program (K1-K3), medium-term results that track progress in achieving the Sustainable Development Goals until 2030, and a long-term impact assessment that assesses the contribution to the strategic goals of Kazakhstan-2050. This multi-time approach ensures that monitoring performs the functions of operational management and strategic planning.

### 4.1 Implementation challenges

Despite the obvious advantages, the implementation of DMNPM faces serious problems that require strategic approaches:

Institutional barriers include resistance from civil servants accustomed to traditional reporting methods, insufficient technical expertise of modern analytical tools, and difficulties with interdepartmental coordination. These tasks require comprehensive change management strategies, including training programs, incentive coordination, and gradual implementation approaches.

Technical limitations are related to the quality of data in existing government systems, outdated IT infrastructure in some departments, and cybersecurity issues for integrated data sharing. Solving these problems requires significant investments in infrastructure and reliable security systems that balance access with protection. Recent systematic reviews identify eight main research topics in the field of digital technology implementation, which shows that technical infrastructure alone is not enough, without removing broader socio-economic barriers (Liu et al., 2025).

The gaps in the regulations are reflected in the lack of standards for collecting and exchanging data, an unclear legal framework for the use of open data, and a lack of proposals for the implementation of artificial intelligence in the government. The resolution requires a comprehensive development of regulatory legal acts that establish specific standards and reporting mechanisms.

### 4.2 Comparative advantages and global applicability

DMNPM offers several advantages that place it for wider international use. The modular architecture allows you to adapt to different institutional contexts and technical capabilities, and relying on widely available tools (Python, Power BI) makes implementation accessible even for governments with limited resources.

The focus of developing countries distinguishes DMNPM from structures for advanced digital ecosystems. The model clearly addresses data fragmentation, resource constraints, and institutional constraints that are often found in the development of contexts, making it more realistic and accessible than idealized structures that require perfect conditions.

The integration orientation emphasizes working with existing systems without the need for a complete replacement, reducing implementation costs and barriers, while allowing the gradual development of capabilities. This approach proves to be more stable than revolutionary changes that often fail due to resource or bandwidth constraints.

The international application potential of the model is especially important because developing countries face similar challenges and can benefit from common implementation experience and adapted solutions, rather than trying to replicate structures for fundamentally different contexts.

Thus, DMNPM does not simply adapt global practices, but integrates them into the specific conditions of Kazakhstan, overcoming challenges characteristic of developing economies. DMNPM can be tailored to other institutional contexts through a four-phase pathway: (1) Readiness assessment (digital maturity, legal framework, data quality), (2) Architectural localization (API mapping, language adaptation, ML calibration), (3) Pilot implementation (2–3 national programs over 12–18 months), and (4) National scaling (wave-based rollout). The adaptation framework (Supplementary Table S2) provides country-specific implementation pathways.

The developed digital monitoring model offers a number of significant advantages that directly address the problems identified in the introduction, such as information delays and data fragmentation (Fountain, 2001; Lindgren et al., 2019). The flexibility and modularity of the DMNPM architecture allow easy addition of new data sources, adaptation of key performance indicators (KPIs), and customization of analytical modules, which corresponds to the principles of adaptive management.

Automation of data collection through API integration (Smart Bridge) and from open sources (Open Data) significantly reduces labor costs, minimizes errors, and enables monitoring in near-real-time, which is critically important for operational decision-making. Comprehensive assessment, including budget efficiency, goal achievement, and socio-economic effect, provides deeper and more comprehensive analysis, consistent with the principles of result-oriented public administration (Osborne and Gaebler, 1992). The inclusion of predictive analytics allows transitioning from problem statement to their prediction, which contributes to proactive risk and resource management. Finally, interactive dashboards and improved data visualization increase transparency and potentially strengthen civic participation.

The contribution of this research to academic theory lies in proposing a detailed conceptual model that integrates various aspects of digital government and analytical capabilities to address specific challenges of monitoring national programs, expanding the existing understanding of Digital M&E, especially in the context of using national platform solutions.

Despite the obvious advantages, the implementation and full functioning of DMNPM are associated with a number of significant limitations requiring a strategic approach. Institutional barriers include inertia and possible resistance to change from civil servants, as well as a shortage of qualified personnel capable of working with modern analytical tools. Interagency disunity and insufficiently developed culture of data-driven decision-making remain serious challenges requiring cultural transformation (Grindle, 2007).

Among technical limitations, the problem of source data quality in existing government information systems (incompleteness, inaccuracy, inconsistency) stands out, which requires significant efforts for their cleaning and validation. Problems with outdated IT infrastructure in some departments and information security issues can also impede full integration and data exchange. Regulatory limitations manifest in the absence of unified standards for data collection and exchange, as well as gaps in legislation regulating the use of open data and analytical tools.

The developed DMNPM has significant potential for adaptation and application in other developing countries, which is due to the similarity of challenges they face: data fragmentation, limited resources, and an acute need to increase transparency and efficiency of government programs (World Bank, 2021; UNDP, 2022). The model, developed considering the specific conditions of Kazakhstan, can serve as a reference framework. The use of publicly available and widespread technical tools (Python, Power BI) makes it relatively inexpensive and accessible even for countries with limited IT budgets. The modular architecture provides flexibility for adaptation to specific data features and institutional structure.

### 4.3 Future research

As a result of this study, several important research priorities appear. Empirical validation through experimental implementations is very important to test the effectiveness of the model in specific situations and identify practical challenges. The study of intercultural adaptation at different levels of management systems and development improves the applicability of the model. The study of technical developments should focus on the framework of artificial intelligence ethics, cybersecurity architecture and interaction standards. Citizen-oriented research should explore optimal interaction mechanisms and the consequences of digital divergence. The implementation of scientific research should take into account change management processes, capacity building approaches, and long-term sustainability requirements.

The experience of Kazakhstan, which is actively implementing digitalization, can become a valuable reference point that will help other countries avoid such mistakes. Future implementations should explore deeper integration with Kazakhstan’s established situational center infrastructure (Janenova, 2018) and incorporate collective intelligence methods that have proven effective in improving government data infrastructures (Lowry et al., 2023). Thus, the DMNPM provides a practical and scientifically based solution for improving the efficiency of monitoring national programs, which is very important for achieving the Sustainable Development Goals and improving the quality of public administration in developing countries.

## 5 Conclusion

This study was devoted to the development and conceptual substantiation of a Digital Model for National Program Monitoring (DMNPM) in the context of digital transformation of public administration in the Republic of Kazakhstan. The identified limitations of traditional monitoring approaches, such as data fragmentation, lack of operativeness, and insufficient focus on results, emphasized the relevance of the proposed solution.

The theoretical contribution of this research lies in expanding the understanding of digital governance capabilities and introducing a comprehensive methodology for evaluating government programs into scientific circulation. The proposed three-level model (K1 - budget efficiency, K2 - goal achievement, K3 - socio-economic effect) goes beyond traditional approaches, offering deeper and multidimensional analysis. We have shown how a mixed research method, combining quantitative data from open sources and specialized APIs with qualitative analysis, allows obtaining a comprehensive picture. Moreover, the work fills gaps in the literature by offering a detailed analysis of Kazakhstan’s experience, which is especially valuable for studying digital transformation in developing countries.

During the research, a conceptual architecture of DMNPM was successfully developed, its key components were described in detail, including input data, collection, processing, analysis and visualization modules, as well as feedback mechanisms. The role and potential of integrating existing government information systems of Kazakhstan (egov.kz, Smart Bridge, Open Data) as a foundation for building this model were substantiated. Demonstration of DMNPM applicability on examples of national programs “Digital Kazakhstan” and “Nurly Zhol,” as well as pilot projects “Zhaily Mektep” and “Auyldyq Densaulyq Saqtau” clearly illustrated the model’s capabilities for ensuring dynamic progress tracking, using predictive analytics for risk forecasting, and increasing transparency.

The scientific contribution of the study lies in the provision of an original conceptual model that systematically combines advanced digital technologies (including predictive analytics based on machine learning) and feedback mechanisms to improve the effectiveness of monitoring national programs. This contributes to filling existing research gaps in the field of holistic models of digital monitoring adapted to the conditions of active development of digital ecosystems, such as Kazakhstan’s.

An empirical analysis of the national projects “Zhaily Mektep” and “Auyldyq Densaulyq Saqtau” revealed the most important success factors in the implementation of digital monitoring systems, including the need to use indirect indicators to accurately quantify goals, ensure the flexibility of models and assess the socio-economic impact. A comparative analysis of the budget execution of Republican projects showed significant discrepancies (from 35.1 to 100%), confirming the need for an integrated approach to monitoring budget execution beyond traditional control.

Practical recommendations for government agencies of the Republic of Kazakhstan include the need for further development of unified standards for data collection and exchange, investments in developing digital competencies of civil servants, as well as creating an appropriate regulatory framework for full implementation and scaling of DMNPM. Active use of the potential of Smart Bridge and Open Data can become a catalyst for transitioning to a qualitatively new level of data-driven public administration.

Prospects for future research include empirical testing and pilot implementation of the developed model in real conditions to confirm its effectiveness and identify practical tasks. Comparative studies with similar initiatives in other countries are also of interest to identify the universal and contextual success factors of digital monitoring.

Ultimately, DMNPM offers not only a technological but also an institutional solution that contributes to the formation of a more transparent, accountable, and effective public administration system, which is critically important for achieving national strategic goals and Sustainable Development Goals.

## Data Availability

The original contributions presented in the study are included in the article/Supplementary material, further inquiries can be directed to the corresponding author.
