# Supplementary material for: Digital model for monitoring national programs: the Kazakhstan experience
Source: Front Artif Intell. 2025 Nov 19;8:1656329. doi: 10.3389/frai.2025.1656329 (PMC12672492; doi:10.3389/frai.2025.1656329)
Supplement: Supplementary file 1 [file Data_Sheet_1.docx]

**Table S1. DMNPM analytical services and methods**

| **Service** | **Method** | **Mathematical foundation** | **Application** | **Execution time*** |
| --- | --- | --- | --- | --- |
| **MultiplierCalculator** | Proprietary methodology | Input-output multipliers (Leontief framework) | Regional economic impact assessment (NTP, OEP, SPP) | 0.5-1 sec |
| **DichotomousAnalysis** | Binary decision trees | 4×4 aggregation matrix | Production and financial potential screening | 0.2-0.5 sec |
| **AHPAnalyzer** | Analytic Hierarchy Process | Eigenvalue decomposition (Saaty, 1980) | Multi-criteria project prioritization | 1-2 sec |
| **RiskAnalyzer** | Multi-factor scoring | Weighted risk aggregation | Risk identification and quantification | 0.5-1 sec |
| **ForecastService** | Monte Carlo simulation | Stochastic modeling (normal/beta distributions) | Probabilistic outcome forecasting | 3-5 sec |
| **PortfolioOptimizer** | Linear Programming | Binary integer optimization (GLPK-MI) | Budget-constrained portfolio selection | 2-4 sec |

* Based on execution_time_ms field in database. See the technical documentation of the system https://bitbucket.org/jack_shepherd/tetra-project-docs-en/src/master/Architecture/database_doc.md. Technical documentation of current system can be found at https://bitbucket.org/jack_shepherd/tetra-project-docs-en/src/master/.

**Table S2. DMNPM Adaptation Framework for Different Country Contexts**

| **Country Type** | **Digital Maturity (UN EGDI)** | **Adaptation Pathway** | **Timeline** | **Key Success Factors** |
| --- | --- | --- | --- | --- |
| Type A: High maturity (e.g., Estonia, Singapore) | EGDI > 0.85 | Direct implementation with advanced ML modules | 12-18 months | API standardization, AI governance frameworks |
| Type B: Medium maturity (e.g., Brazil, Philippines) | EGDI 0.65-0.85 | Phased rollout: pilot → scale. Start with statistical analytics, add ML as data matures | 18-36 months | Interagency coordination, capacity building, data quality improvement |
| Type C: Developing (e.g., Kenya) | EGDI 0.50-0.65 | Lightweight version: focus on data aggregation + basic dashboards first. ML as long-term goal | 24-48 months | Donor support, infrastructure investment, regulatory framework development |

The DMNPM adaptation path follows a structured four-phase methodology based on a systematic analysis of digital government maturity frameworks and implementation principles. The initial readiness assessment phase typically lasts three to six months and includes a comprehensive diagnostic, including a digital infrastructure audit, a legal gap analysis, stakeholder mapping, and a baseline data quality assessment. This phase employs standardized instruments such as the UN E-Government Development Index (EGDI) and World Bank GovTech Maturity Index to establish country-specific benchmarks and identify critical enablers or barriers to implementation.

Following the diagnostic, the architecture localization phase lasts six to twelve months and focuses on technical adaptation activities. This includes mapping existing government information systems to DMNPM modular components, customizing key performance indicators to align with national strategic priorities, creating data management protocols that align with local regulations, and developing professional development training programs tailored to the professional skills of civil servants. Particular attention during this phase is paid to API compatibility issues and the creation of backup mechanisms for manual data integration in cases where automated pipelines encounter infrastructural limitations.

The pilot implementation phase represents controlled deployment across two to three strategically selected national programs over twelve to eighteen months. Selection criteria prioritize programs with established monitoring routines, diverse geographic coverage, and executive champion commitment. During this phase, the functionality of the data pipeline is validated, the accuracy of the analytical modules is tested, and user interfaces are iteratively refined based on stakeholder feedback. Finally, the national scale-up phase organizes a phased rollout across all strategic programs over eighteen to thirty-six months, using wave deployment strategies that combine rapid expansion with quality assurance. Critical success factors throughout all phases include ongoing leadership support at the ministry level, dedicated change management teams with cross-functional expertise, and ongoing engagement with civil society to validate transparency mechanisms and ensure accountability meets citizen expectations.

**Table S3. Comparative benchmarking of digital monitoring systems**

| **Characteristic** | **Estonia (X-Road)** | **South Korea (DPG)** | **Canada (GC InfoBase)** | **DMNPM (Current/Roadmap)** |
| --- | --- | --- | --- | --- |
| Architecture | Federated data exchange layer | Centralized platform | Hybrid (federated + portals) | Modular integration layer |
| Data integration | Seamless (mature ecosystem) | Medium (legacy silos remain) | High (standards-driven) | High (API + ETL pipelines) |
| AI/Analytics | Limited (rule-based automation) | Active (chatbots, NLP) | Expanding (exploratory dashboards) | Foundational statistical + ML-ready architecture |
| Transparency | Partial (X-Road logs public) | Good (open data portals) | Good (GC InfoBase public) | Two-way feedback + open dashboards |
| Cost efficiency | High (reusable infrastructure) | Medium (requires skilled staff) | Medium (multi-vendor complexity) | High (open-source stack, modular) |
| Adaptability to developing contexts | Low (requires mature digital ecosystem) | Medium (infrastructure-intensive) | Medium (resource-dependent) | High (designed for fragmented data environments) |
